# Virtual Cancer Care During the COVID-19 Pandemic and Beyond: A Call for Evaluation

**DOI:** 10.2196/24222

**Published:** 2020-11-24

**Authors:** Oren Hannun Levine, Michael McGillion, Mark Levine

**Affiliations:** 1 Department of Oncology McMaster University Hamilton, ON Canada; 2 Hamilton Health Sciences - Juravinski Cancer Centre Hamilton, ON Canada; 3 School of Nursing McMaster University Hamilton, ON Canada

**Keywords:** care, patient-physician relationship, patient-centered care, oncology care delivery, virtual visits, telehealth, virtual care, cancer, oncology, evaluation, COVID-19

## Abstract

The interplay of virtual care and cancer care in the context of the COVID-19 pandemic is unique and unprecedented. Patients with cancer are at increased risk of SARS-CoV-2 infection and have worse outcomes than patients with COVID-19 who do not have cancer. Virtual care has been introduced quickly and extemporaneously in cancer treatment centers worldwide to maintain COVID-19–free zones. The outbreak of COVID-19 in a cancer center could have devastating consequences. The virtual care intervention that was first used in our cancer center, as well as many others, was a landline telephone in an office or clinic that connected a clinician with a patient. There is a lack of virtual care evaluation from the perspectives of patients and oncology health care providers. A number of factors for assessing oncology care delivered through a virtual care intervention have been described, including patient rapport, frailty, delicate conversations, team-based care, resident education, patient safety, technical effectiveness, privacy, operational effectiveness, and resource utilization. These factors are organized according to the National Quality Forum framework for the assessment of telehealth in oncology. This includes the following 4 domains of assessing outcomes: experience, access to care, effectiveness, and financial impact or cost. In terms of virtual care and oncology, the pandemic has opened the door to change. The lessons learned during the initial period of the pandemic have given rise to opportunities for the evolution of long-term virtual care. The opportunity to evaluate and improve virtual care should be seized upon.

## Introduction

The COVID-19 pandemic has catapulted virtual care into the forefront of oncology practice [1-7]. The interplay of virtual care and cancer care in the context of the pandemic is unique and unprecedented [1-7]. Patients with cancer are at increased risk of SARS-CoV-2 infection because of immunosuppression [8,9] and frequent visits to cancer centers for therapy, which potentially increases their risk of contracting and transmitting COVID-19 [9]. Furthermore, the outcomes of patients with cancer and COVID-19 are likely worse than those of patients with COVID-19 who do not have cancer [10-14]. The introduction of virtual care during the onset of the pandemic was an emergency strategy for maintaining cancer centers as COVID-19–free zones to avoid any potential interruption in treatments.

In this commentary, virtual care is defined as an interaction between clinicians and patients that occurs remotely through communication or information technologies with the aim of facilitating or maximizing the quality and effectiveness of patient care [15,16]. During the onset of the pandemic, a landline telephone in an office or clinic was first used in our cancer center to conduct consultations and follow-up assessments, share test results with patients and families, and have delicate and difficult conversations. The use of telephone landlines was our first immediate option for remote care during the pandemic crisis. This experience stimulated our thoughts on virtual care and our need to increase capacity in this regard. Initially, there was limited video connectivity in cancer centers. However, this is now changing, and the pandemic has allowed for virtual care approaches to evolve.

Virtual care has been introduced quickly and has featured extemporaneous implementation under time pressure [1-7]. It is anticipated that there will be pressure to continue virtual care in oncology because of its efficiency and potential to cut costs [17]. However, there is a lack of virtual care evaluation from the perspectives of patients and oncology health care providers. Herein, we consider the impact of the virtualization of oncology practices with respect to a number of factors. Based on our recent experience with virtual care, albeit mostly telephone-based care, we highlight opportunities to evaluate models of care in oncology practices that incorporate any virtual technology. The factors that we consider are organized according to the National Quality Form framework for the assessment of telehealth in oncology. This includes the following 4 domains of assessing outcomes: experience, access to care, effectiveness, and financial impact or cost [18] (Table 1).

**Table 1:** National Quality Forum telehealth measurement framework.

Factors	Domains^a^			
	Access to care	Financial impact or cost	Experience	Effectiveness
Patient rapport			+	
Patient frailty			+	
Delicate conversations			+	
Multidisciplinary care	+			
Role of the nurse	+			
Resident education				+
Patient safety			+	+
Technical effectiveness				+
Privacy				+
Operational effectiveness		+		+
Resource utilization		+		

^a^The domain related to a factor.

## Experience

### Patient Rapport

Establishing a strong rapport with patients is important for building trust [19]. A patient’s first visit to a cancer center is often the most important for building strong clinician-patient relationships [19]. Diagnoses, prognoses, and treatment options are usually addressed in the first consultation visit. Furthermore, the physical examination can impact the care plan, and thorough assessments may contribute to a sense of trust with medical care. With remote care, it can be more challenging to establish patient rapport. Strong rapport is helpful for identifying when a patient’s status has changed (eg, cancer spread) and providing compassionate care [19,20]. Methods for optimizing the sense of connectedness between patients and care providers during virtual care requires further study.

### Patient Frailty

Many patients with cancer are older adults who have other comorbid medical illnesses. Frailty is not an illness; it is a syndrome that combines the effects of natural aging with the outcomes of multiple long-term conditions, such as the loss of fitness and reserves [21]. Chemotherapy is often associated with toxicity, which can sometimes be life-threatening, and toxicity tends to increase with age. However, there are older patients whose physical conditions are robust. It is important to be able to assess the frailty of the patient to avoid the risk of excessive toxicity and undertreatment. The reliability of remote frailty assessments requires exploration.

### Delicate Conversations

Bad news conversations can be very difficult during remote care, especially over the phone [22,23]. Even when using video-based technology, it may not be possible to pick up on body language and visual cues to gauge how a patient is receiving information. The parameters of video-based communication can limit direct eye contact and leave room for miscommunication and the indeterminacy of one’s intent [24]. Thus, it remains unclear whether visits scheduled for potentially sensitive conversations should be done virtually or in person. If such conversations are done in person during the time of a pandemic, the patient must arrive alone for what may be a difficult and anxiety-provoking experience. Assessing patient experience may help define a reasonable standard.

## Access to Care

### Multidisciplinary Care

Multidisciplinary clinics are an important part of specialized oncology care at any major cancer center [25]. Surgical, medical, and radiation oncologists assess patients together in order to make a treatment recommendation. Usually, this requires multiple people in 1 room and violates physical distancing recommendations. Virtual technology allows multiple specialists to interact with a patient at the same time, but it can be cumbersome and logistically challenging. Patient and care provider satisfaction is an important measure for ensuring that multidisciplinary care is sustainable in a virtual model.

### Role of the Nurse

In many cancer centers, the model of nursing is a shared-care model between oncologists and nurses. Nurses have many responsibilities regarding patient care, including symptom assessment, health education, and triaging calls regarding treatment toxicity and the psychologic, emotional, and social aspects of care [26]. There is potential for the nurse’s role to be marginalized due to virtual care. We see a need for the in-depth evaluation of the impact of virtual care on the supportive and relational aspects of nursing work.

## Effectiveness

### Resident Education

Another challenge is incorporating medical student and postgraduate resident education into virtual practice. Traditionally, in clinics, a resident enters a patient examining room to take an illness history and perform a physical examination. Afterward, they leave the room and confer with the staff oncologist, which is an opportunity for on-the-fly teaching. Upon returning to the patient together with the staff oncologist, there is a chance for bedside teaching. This process cannot be performed with remote care. Recently, there has been a shift toward competency-based residency education, which emphasizes direct observation and feedback [27,28]. Video calls may allow for the observation of a resident’s communication skills. However, whether this is sufficient to establish a trainee’s competence for clinical practice requires validation.

### Patient Safety

It is routine for a patient to be weighed at every visit. Weight can be an important clue for determining changes in health status and the need to change drug dosing. Standardized symptom assessments are completed before visiting the clinician. These assessments also serve as a screen for important changes in health status. However, the uptake of online symptom screening has been inconsistent. Without these early warning signs, are patients more likely to experience toxicity? Health services research could help elucidate this question.

### Technical Effectiveness

It is important to keep in mind that many patients do not have access to video calling software, high-speed internet, or email [29-31]. Furthermore, the patient’s prior experience with technology may affect the success of virtual encounters [29-31]. Occasionally, sound quality, language barriers, and hearing impairment make it difficult to determine if information has been understood correctly. Other barriers to virtual communication include somnolence and confusion from chemotherapy, supportive medications (eg, narcotics), or advanced cancer [32].

### Privacy

Patients and care providers must trust that the information being transmitted during care is private and secure [30,31,33]. When health care practices are conducted virtually and all information is transferred electronically, the situation becomes more complex. There remains much to be learned about implementing and scaling virtual care in oncology per the Hospital Level 7 integration standards for seamless and cybersecure hospital-to-home connection [34]. The best practices for implementing virtual care models that measurably preserve patients’ and families’ privacy and ensure the security of data throughout the virtual care process are paramount [34].

## Financial Impact or Cost

### Operational Effectiveness

Patient convenience and clinical service-related satisfaction may be enhanced through virtual care, as costly parking fees and lengthy periods in waiting rooms can be avoided. Physician reimbursement was an issue during the beginning of the pandemic due to the rapid implementation of virtual care in oncology, but this has been addressed [33]. From the clinician’s perspective, follow-up visits may be shorter, allowing for more patient assessments. However, if a patient is unavailable, is time lost through repeated attempts to contact that patient? Whether virtual care in oncology is more efficient than in-person care remains unknown, but this should be studied [33,35].

### Resource Utilization

If patients perceive a lack of access to cancer centers when urgent in‑person assessments are needed, they may resort to visiting the emergency department for symptom complaints or treatment toxicity. Administrative data should be scrutinized to assess the impact of virtual care on acute care resource utilization [35].

## Conclusion

In terms of virtual care and oncology, the COVID-19 pandemic has opened the door to change. The lessons learned during the initial period of the pandemic have given rise to opportunities for the evolution of long-term virtual care. It would be unfortunate not to learn from our experiences through thoughtful and scholarly assessment. Assessment measures should span the areas of experience, access to care, effectiveness, and financial impact or cost. The opportunity to evaluate and improve virtual care should be seized upon.
